# Construction of the Single‐Cell Landscape of Hashimoto's Thyroiditis Tissue and Peripheral Blood by Single‐Cell RNA Sequencing

**DOI:** 10.1002/iid3.70153

**Published:** 2025-02-11

**Authors:** Kaiyu Song, Xiaojie Wang, Wenjie Yao, Yuantao Wang, Qinling Zhang, Yuxiao Tang, Yakui Mou, Xicheng Song, Jin Zhou

**Affiliations:** ^1^ Department of Endocrinology Yantai Yuhuangding Hospital of Qingdao University Yantai Shandong China; ^2^ Department of Otorhinolaryngology, Head and Neck Surgery, Affiliated Hospital of Weifang Medical University, School of Clinical Medicine Weifang Medical University Weifang Shandong China; ^3^ Department of Endocrinology, Second Clinical Medical College Binzhou Medical University Yantai Shandong China; ^4^ Department of Otorhinolaryngology, Head and Neck Surgery, Yantai Yuhuangding Hospital Qingdao University Yantai Shandong China; ^5^ Department of Otorhinolaryngologic Diseases Yantai Shandong China; ^6^ Department of Endocrinology and Foot & Ankle Surgery Yantai Shandong China

**Keywords:** hashimoto's thyroiditis, immune cells, pathogenesis, single‐cell sequencing, transcription factors

## Abstract

**Background:**

Hashimoto's thyroiditis (HT) is the most common organ‐specific autoimmune disease, and its etiology may be related to genetic, environmental, and epigenetic factors. However, its exact pathogenesis remains elusive.

**Methods:**

In this study, single‐cell transcriptomic sequencing and bioinformatics analysis were performed on the thyroid tissues of six HT patients, peripheral blood mononuclear cells (PBMCs) of four HT patients, and normal thyroid tissue of one healthy control. A panoramic single‐cell atlas of HT was constructed to explore changes in the abundance of different cell subsets in the states of the disease.

**Results:**

A single‐cell atlas of HT was constructed, and eight cell types were defined based on the marker genes. Subsequent clustering analysis of T cells, B cells, myeloid, and thyroid follicular cells revealed that the abundance rates of the CD8^+^T_CCL4L2, B_MEF2B_BCL6, Mac_APOE, Mac_IL1B, and TFC_PAX8_NKX2‐1 subgroups were elevated in thyroid tissues of HT patients. However, the abundance rate of the NKT_KLRD1_KLRC2 subgroup was risen in the PBMCs of HT patients. Ig‐producing plasma cells were specifically enriched in the B‐cell subgroup.

**Conclusion:**

The present study further validated the role of immune cells in the pathogenesis of HT at the cellular level. In addition, a new cell subset B_MEF2B_BCL6 was found. It could be speculated that MEF2B mainly transactivates the expression level of the transcriptional repressor BCL6, leading to the development of HT. A new cell subset TFC_PAX8_NKX2‐1 was also identified, in which the specific transcription factors PAX8 and NKX2‐1 were highly expressed in HT tissues.

## 1 Introduction

Hashimoto's thyroiditis (HT), also referred to as chronic lymphocytic thyroiditis or autoimmune thyroiditis, along with Graves' disease (GD), constitutes a significant category of autoimmune thyroid diseases (AITD) [1]. The overall prevalence of HT is approximately 7.5%. In females, the prevalence is about 17.5%, while in males, it is around 6.0% [2]. These figures can vary based on geographic region. Notably, the condition is significantly more common in females, with only about 10% of patients being male [3]. It is increasingly recognized that a combination of genetic, environmental, and immune factors plays a critical role in the development of HT. Individuals predisposed genetically may experience triggers from environmental factors, activating CD4^+^ T cells that target thyroid autoantigens. These activated CD4^+^ T cells serve to stimulate B cells, leading to the production of autoantibodies against thyroid antigens. At the same time, the pro‐inflammatory cytokines and chemokines produced by activated CD4^+^ T cells further amplify and prolong the autoimmune response by prompting thyroid cells to release pro‐inflammatory substances [4]. Additionally, they can recruit CD8^+^ cytotoxic T cells, directly damaging thyroid cells. Histopathological evaluations of HT reveal diffuse infiltration of lymphocytes, varying degrees of fibrosis, and the formation of lymphoid follicles with germinal centers (GC) [5]. The GC is a key region for B cell clonal expansion and antibody affinity maturation within lymphoid follicles. B cells undergo somatic hypermutation, selecting those with a high affinity for antigens. Rapidly dividing centroblasts in the dark zone migrate to the light zone, where they become centrocytes and express more immunoglobulin. After affinity maturation and isotype switching, some of these cells differentiate into antibody‐secreting plasma cells, which then exit the germinal center to enter peripheral tissues [6]. Thyroid autoantibodies, particularly TgAb and TPOAb, can directly harm thyroid cells by blocking TSH receptors or through mechanisms like antibody‐dependent and complement‐dependent cell‐mediated cytotoxicity [7]. This cascade of immune activity can ultimately lead to hypothyroidism in patients with HT. In summary, the hallmark features of HT are characterized by local lymphocyte infiltration, the production of autoantibodies, and varying degrees of damage and dysfunction in thyroid cells. Current HT's therapeutic strategies often wait for HT to progress to hypothyroidism before administering thyroid hormone supplementation. This approach overlooks the inflammatory and immune mechanisms involved in the development and progression of HT. To address this gap, our research hopes to contribute to developing strategies that target inflammation and immunity. In the present study, we constructed a comprehensive single‐cell map of HT, utilizing single‐cell transcriptomic data obtained from thyroid tissues of six HT patients, PBMCs of four HT patients, and normal thyroid tissue from one healthy control. Variations in the levels of different cell subsets were assessed, providing a scientific foundation for comprehending the pathogenesis of HT and advancing promising treatments for the disease.

## 2 Materials and Methods

### 2.1 Human Samples

**Figure 1:**
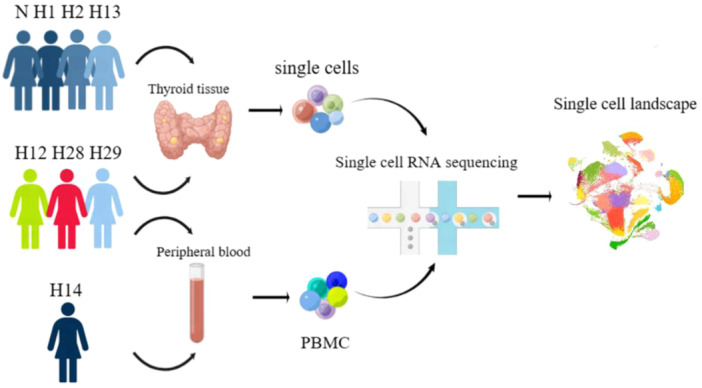
Schematic illustration of workflow from sampling to sequencing.

This study incorporated thyroid lesion tissues from six HT patients, PBMCs from four patients, and normal thyroid tissue adjacent to the nodule from a healthy control. Notably, two patients and one healthy control, who participated in the study, consented to a comprehensive library and sequencing plan involving all research procedures. The study was approved by the Ethics Committee of Yantai Yuhuangding Hospital (Yantai, China; Approval No. 2022‐278). Thyroid tissue samples from patients who have undergone surgery and paramodular thyroid tissue samples from those with suspicious thyroid malignant nodules (control group) were obtained for single‐cell transcriptomic sequencing. The data of the remaining research subjects were extracted from the Gene Expression Omnibus (GEO) database (GEO Accession No. HRA001684) [8]. Thyroid tissues and PBMCs were obtained from five patients (HT12, HT13, HT14, HT28, and HT29). In this study, no PBMC of HT13 was obtained, and the cellular content of scRNA‐seq data of HT14 thyroid tissue was insufficient for subsequent analysis. Further clinical data are presented in Supporting Information S1: Tables 1 and 2. Finally, the abovementioned data were integrated for data analysis (Figure 1). It is noteworthy that the units of FT4 differ due to variations in the methods of measurement, with a conversion factor of 1 pmol/L = 12.87 ng/dL. Furthermore, in patients with papillary thyroid carcinoma combined with HT, tissue sampling was conducted at a location at least 5 cm away from the cancerous lesion, which was considered as a normal tissue rather than paraneoplastic (Supporting Information S1: Tables 1 and 2).

### 2.2 Single‐Cell Transcriptomic Sequencing

The 10× Genomics platform is a commonly employed single‐cell transcriptomic sequencing platform. It incorporates magnetic beads, containing read1 primers, cell barcodes, and unique molecular identifiers (UMIs) with single‐cell suspensions, involving reverse transcriptase and nucleotides. This promotes binding the cells to the magnetic beads, which are subsequently quickly trapped and encapsulated in oil droplets at the proper rate and collected into a test tube. The magnetic beads are suspended in cell lysis buffer, contained within oil droplets. Within these droplets, cell lysis occurs, releasing mRNA, which is subsequently reversely transcribed into DNA using reverse transcriptase. Following the breakage of the oil droplets, the cDNA is extracted for subsequent amplification sequencing. Each magnetic bead is uniquely identified by its cell barcode, ensuring that the same cell barcode corresponds to the same cell. Moreover, each bead carries a diverse series of UMIs. Each UMI represents an mRNA molecule, and the expression level of a gene can be determined by the number of UMIs associated with the same mRNA molecule. Through this technology, high‐throughput single‐cell transcriptomic sequencing can be accomplished.

Sample preparation and cDNA library construction in this study were performed in accordance with the instructions provided by the manufacturer of 10× Genomics Single Cell 3'V3.1 kit. The cDNA product and library concentration were determined using the Qubit 4.0 fluorescence quantitative instrument, and the cDNA library insert size was determined via the QSEQ 400 biological analyzer. Subsequently, the sample library was sequenced by the Novaseq 6000 instrument on the Illumina platform. Following recognition by the CASAVA base, the raw imaging file was converted into a sequence file and stored in the FASTQ format. Finally, the sequencing data were compared and quantified using 10× Genomics Cell Ranger software.

### 2.3 Construction of Single‐Cell Atlases

The standardized processing of single‐cell data in this study was conducted using the RunSeuratNorm and sctransform functions from the Seurat package of the R programming language. The sctransform function employs regularized negative binomial modeling to analyze single‐cell UMI expression data, eliminating variations due to sequencing depth while preserving true biological heterogeneity.

The single‐cell atlas construction was conducted based on the RunSeurat, utilizing the default parameters of the Seurat package in R programming language for cell clustering [9]. To integrate all single‐cell data, the IntegrateData function was employed, while the FindClusters function was utilized for clustering. The results of the clustering were then uniformly visualized using the Uniform Manifold Approximation and Projection (UMAP) algorithm. The FindAllMarkers function in the Seurat package was utilized to identify the specific expression gene for each cell cluster, and *p* < 0.05 was considered statistically significant.

The SingleR package in R programming language was applied for cellular annotation. SingleR utilizes a cell sample with a known label type as a reference data set to label and annotate cells in the test data set that are similar to the reference. This approach identifies cell clusters as specific cell types.

### 2.4 Differential Gene Expression Analysis

Differential expression analysis is essential to identify significantly differentially expressed genes (DEGs) among different subpopulations or cell groups. In this study, the FindMarkers function in the Seurat package was utilized to perform the differential expression analysis. Differences in the expression levels of genes between the control group and HT cell subsets were identified, and *p* < 0.05 was indicative of statistical significance.

### 2.5 Subgroup Analysis of Cells

A key objective of single‐cell transcriptomic sequencing of data is to identify cell subpopulations, mainly representing distinct cell types, under specific conditions or tissues, thereby uncovering cellular heterogeneity. In this study, cell sub‐clustering was carried out using the Seurat package in R programming language, and RunSubCellDE enabled further subcluster analysis of each subgroup. The marker genes expressed in each subgroup were identified using the FindAllMarkers function, and the cell subgroups were subsequently classified based on the most abundantly expressed marker genes [10].

### 2.6 Functional Enrichment and Gene Enrichment Analysis

To further explore the biological processes and pathways associated with dysregulated genes, the ClusterProfiler package [11] in R programming language was utilized to perform Gene Ontology (GO) and the Kyoto Encyclopedia of Genes and Genomes (KEGG) pathway enrichment analyses with default parameters. *p* < 0.05 was indicative of a significant difference.

In addition, gene set enrichment analysis (GSEA) of DEGs was conducted using GSEA2‐2.2.4 (Java) software. Using the c5.bp.v6.2.symbols.gmt and c2.cp.kegg.v6.2.symbols.gmt from the MsigDB V6.2 database as the background gene sets, the analysis aimed to uncover potential biological properties.

### 2.7 Single‐Cell Trajectory Analysis

In several biological systems, cells display a continuous spectrum of states and undergo transitions among various cellular states. To better analyze these dynamic processes within cells, pseudo‐time sequences can be utilized. A pseudo‐time sequence is the order of cells along the trajectory of a continuous developmental process in a system, and it allows identification of cell types in the beginning, middle, and end states of the trajectory. In this study, cell developmental trajectories were constructed using RunMonocle2 and RunMonocle3 packages in R programming language. The application of the Monocle2 R package enables simulation of the developmental trajectory of immune cells in the disease microenvironment, undergoing evolutionary reprogramming. The developmental trajectory of HT was reconstructed using the Monocle3 R package [12].

### 2.8 Gene Regulatory Networks (GRNs)

Using pySCENIC, a Python module, GRNs were analyzed and reconstructed that centered on transcription factors (TFs) [12] to identify the internal transcriptional regulatory drivers of HT. The single‐cell regulatory network inference and clustering (SCENIC) [13] provides an important biological perspective on the mechanisms driving cellular heterogeneity by inferring GRNs and identifying cellular states based on single‐cell expression profiles.

The workflow is initiated by describing the input single‐cell expression level profile matrix, followed by the use of regression methods to infer co‐expression modules and identify indirect targets based on the discovery of cis‐regulatory patterns. AUcell was subsequently used to quantify the activities of these regulators by enriching and scoring the target genes of the regulators to calculate regulon activity scores (RASs). The single‐cell data were further refined using the RAS matrix, and RSSs were calculated based on the Jensen‐Shannon divergence to identify cell cluster‐specific regulators. The most specific and important regulons were then mapped to single‐cell cluster profiles and validated using massively parallel sample sequencing (Seek database). Finally, connection specificity index (CSI) matrix was calculated, and regulators were hierarchically clustered according to their CSI values to identify regulatory modules. The relationships between these modules and regulators were subsequently visualized using the ComplexHeatMap package in R programming language [14].

## 3 Results

### 3.1 Single‐Cell Atlas of HT

**Figure 2:**
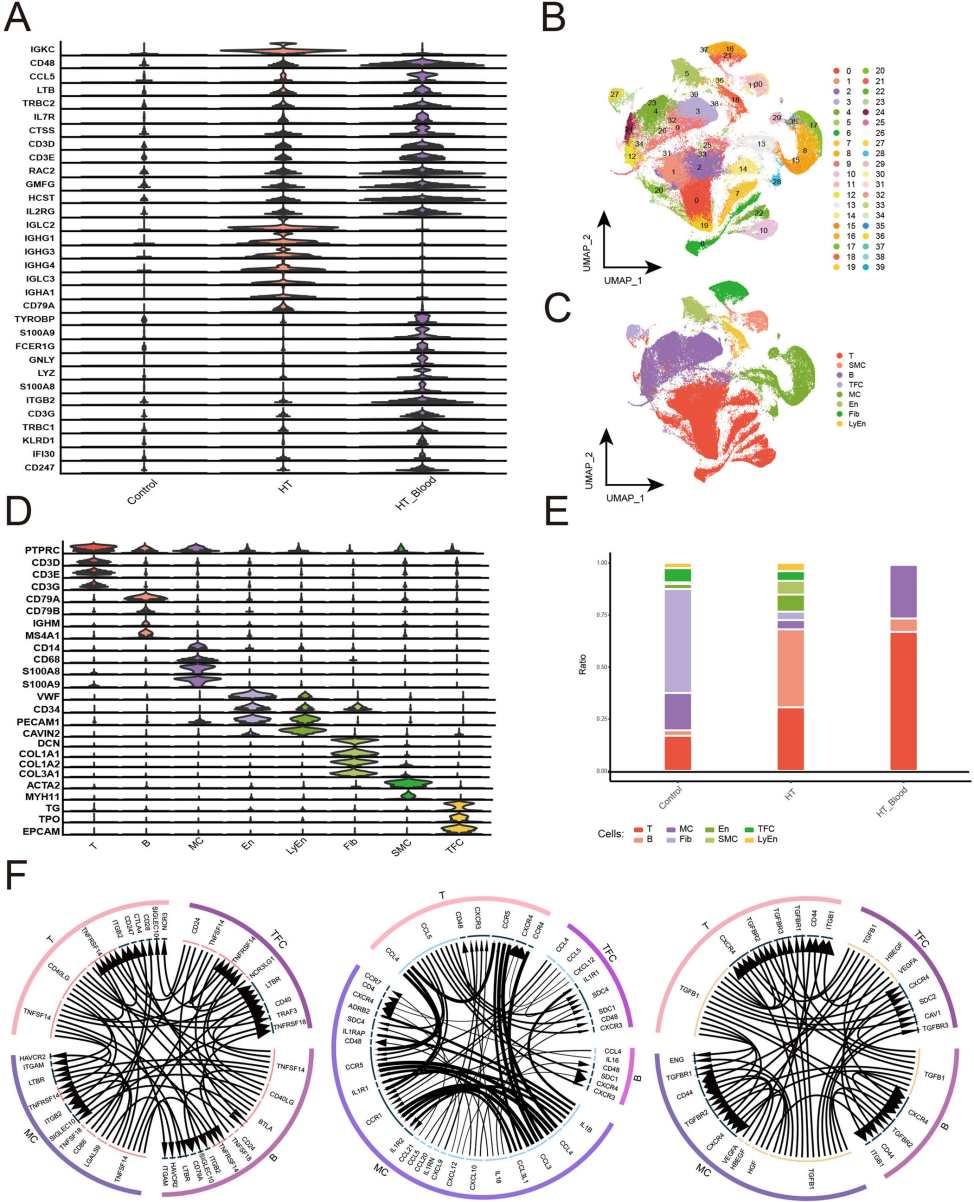
Creating a comprehensive single‐cell atlas of HT: (A) Identification of immune‐related genes associated with HT. (B) Utilizing a total of 142,745 cells and identification of 40 clusters to construct a panoramic single‐cell atlas of HT. (C) Grouping 40 clusters into distinct cell types based on known marker genes, with each color representing a different cell type. (D) Examining the expression levels of marker genes in each cell cluster. (E) Exploration of changes in various cellular fractions in control tissue, HT patient tissues, and peripheral blood mononuclear cells. (F) Investigating interactions among T cells, B cells, MCs, and TFCs in the checkpoint (left), cytokine (middle), and growth factor (right).

Data of immune‐related genes were downloaded from the ImmPort database (http://www.immport.org/shared/), and 2483 immune‐related genes were finally obtained. After removing duplicates, a total of 1793 genes were obtained, of which 93 were found to be associated with HT. The violin diagram shows a subset of immune‐related genes associated with HT (Figure 2A). In addition, to explore the single‐cell global landscape of HT, single‐cell transcriptomic sequencing was carried out, and 142,745 cells were totally identified. Dimensionality reduction with UMAP and graph‐based clustering were then employed to construct a single‐cell atlas, and 40 clusters were identified (Figure 2B). Marker genes were utilized to define the cell clusters as cell types (Figure 2C), T cells, B cells, myeloid cells (MCs), thyroid follicular cells (TFCs), fibroblast cells (Fibs), endothelial cells (ECs), lymphatic endothelial cells (LECs), and smooth muscle cells (SMCs). Expression levels of the cell marker genes in the corresponding cell‐type clusters were confirmed (Figure 2D). Subsequently, the ratios of various cell types among the thyroid tissues of HT patients, PBMCs of HT patients, and thyroid tissue of a healthy control were compared (Figure 2E). It was found that the number of T cells and MCs significantly increased in the PBMCs of HT patients, and the number of B cells was significantly elevated in the thyroid tissues of HT patients. TFCs were found only in the thyroid tissues, and their count was reduced in thyroid tissues of HT patients compared with that in the healthy control, suggesting that the occurrence of HT could be associated with infiltration of immune lymphocytes and destruction of TFCs. Furthermore, the interaction among the aforementioned four cells was investigated by constructing a regulatory network, encompassing checkpoints, cytokines, and growth factors in HT (Figure 2F).

### 3.2 The Role of T Cells in HT

**Figure 3:**
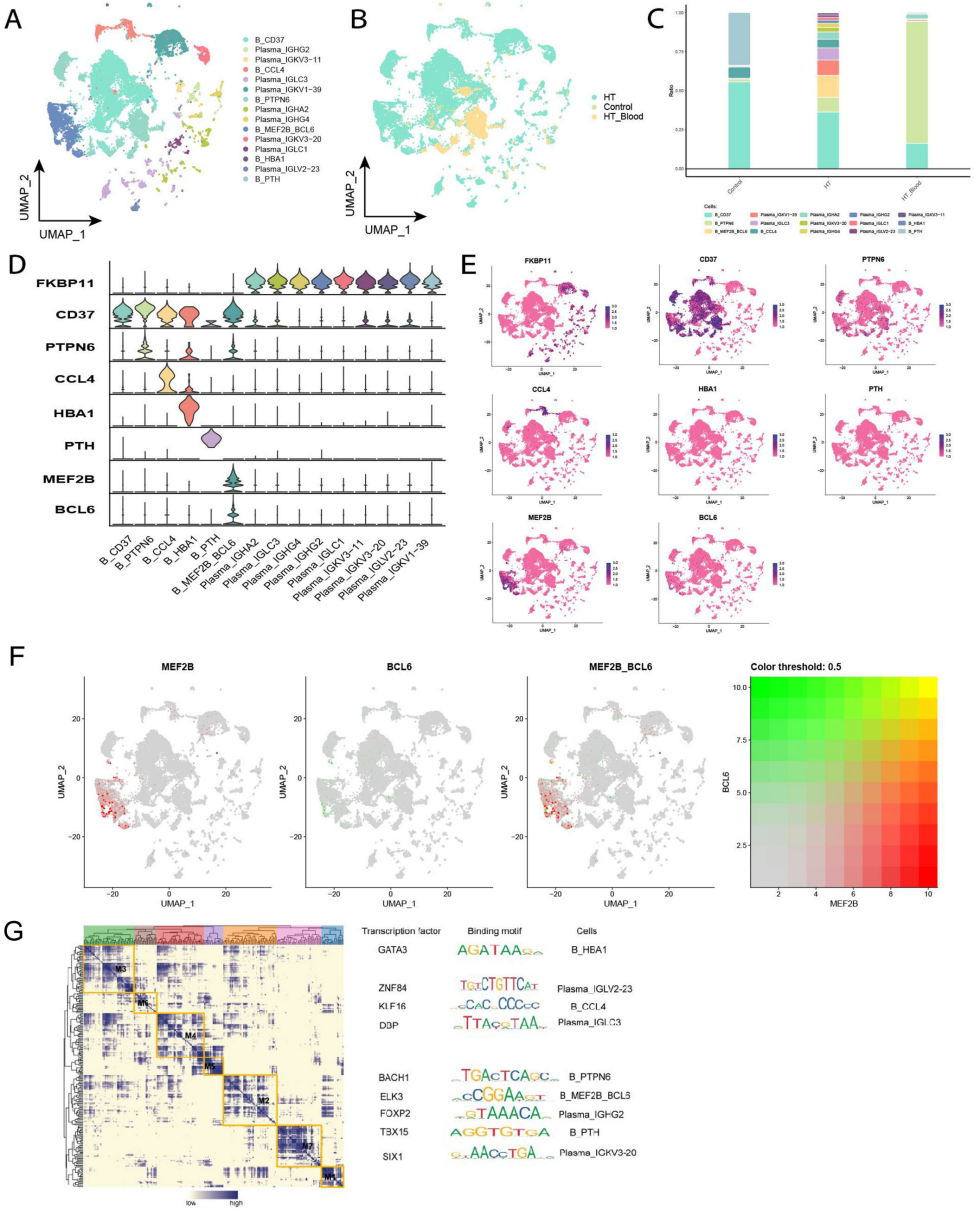
Single‐cell atlas of T cells in HT. (A) Single‐cell profiles of T‐cell subpopulations. Each color represents a different subpopulation. (B) Single‐cell profiles of T cells based on sample source. (C) Differences in abundance of T cell subpopulations in different subgroups. (D) Expression levels of marker genes in each subpopulation. (E) Single‐cell profiles of marker gene expression in each subpopulation. (F) Co‐expression modules of transcription factors in T cells from HT patients. Left: Identification of regulatory modules based on their CSI matrices; middle: binding patterns of representative transcription factors in the modules; right: cellular subpopulations where the transcription factors are located. (G) UMAP single‐cell atlas mapping the specific GRNs of T cell subpopulations.

After reaggregation of T cells, five clusters were totally obtained and mapped in the single‐cell atlas according to different sample sources (Figure 3A,B). In particular, CD4^+^ T cells, CD8^+^ T cells, natural killer T (NKT) cells, and naive T cells were identified based on the expression levels of the CD4 and CD8 genes. It was found that the CD8^+^T_CCL4L2 subpopulation more significantly varied in the tissues of HT patients, and the NKT_KLRD1_KLRC2 subpopulation exhibited a significant variation in the PBMCs of HT patients (Figure 3C). As described earlier, T lymphocytes can directly damage TFCs through cytotoxic effects, as well as recruiting inflammatory cells to infiltrate thyroid tissue by producing relevant inflammatory cytokines, resulting in destruction of TFCs and contributing to the pathogenesis of HT. It could be hypothesized that specific genes expressed in the abovementioned subpopulations of T lymphocytes could be involved in the pathogenesis of HT. All the marker genes were specifically expressed in the corresponding specific subgroup (Figure 3D,E). In addition, GRNs of T cell subpopulations were constructed and the regulators were hierarchically clustered according to the CSI values. The results indicated that TFs were clustered in ARID5B, SOX17, ERG, IRF9, and KLF3, which were categorized into four modules (Figure 3F). These modules, in turn, regulated the expression levels of T cell‐specific genes in HT tissue (Figure 3G).

### 3.3 B_MEF2B_BCL6 Was Enriched in HT Tissue

**Figure 4:**
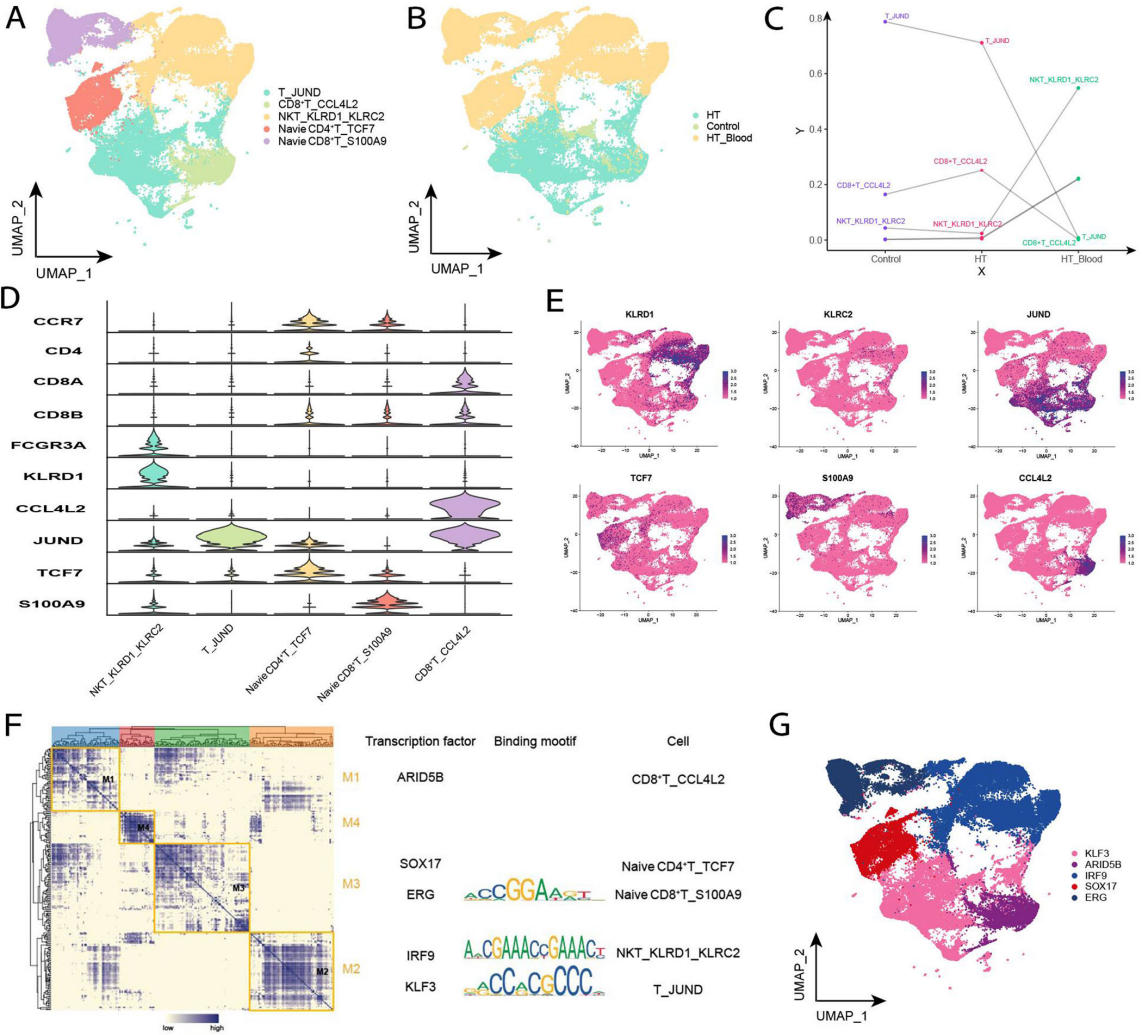
Single‐cell atlas of B cells in HT. (A) Single‐cell profiles of B‐cell subpopulations. Each color represents a different subpopulation. (B) Single‐cell profiles of B cells based on sample source. (C) Composition and variation of B cell subpopulations in different subgroups. (D) Expression levels of marker genes in each subpopulation. (E) Single‐cell profiles of marker gene expression in each subpopulation. (F) Single‐cell profiles showing co‐expression of *MEF2B* gene in relation to *BCL6* gene. (G) Co‐expression modules of transcription factors in B cells from HT patients. Left: Identification of regulatory modules based on their CSI matrices; middle: binding patterns of representative transcription factors in the modules; right: cellular subpopulations where the transcription factors are located.

Subpopulation analysis of B cells according to the annotation method revealed a total of 15 cell subpopulations (Figure 4A). FKBP11 is specifically localized in antibody‐producing plasma cells [15], thus, B cells expressing FKBP11 were identified as plasma cells. Notably, nine clusters were classified as plasma cells, and further variations in B cell subpopulations from different sample sources were evident in the single‐cell profiles (Figure 4B). A significant increase was found in Ig‐producing plasma cell subpopulations in HT patients, as well as a more significant changes in B cell subpopulations that were double‐positive for MEF2B and BCL6 genes (Figure 4C). Expression levels of specific genes in each subpopulation are illustrated in violin maps and single‐cell atlases (Figure 4D,E). Additionally, MEF2B and BCL6 genes were co‐expressed (Figure 4F), suggesting that these specific genes could be involved in HT pathogenesis by influencing the function of B cells. The relationship between MEF2B and BCL6 genes and the transcription factor ELK3 was also explored (Figure 4G). Three genes were expressed in B cells, and MEF2B [16] and BCL6 [17] genes and the transcription factor ELK3 [18] have previously exhibited to have antiapoptotic effects. It could be hypothesized that the three genes could act together on B cells to promote B cell infiltration in HT.

### 3.4 The Role of MCs in HT

**Figure 5:**
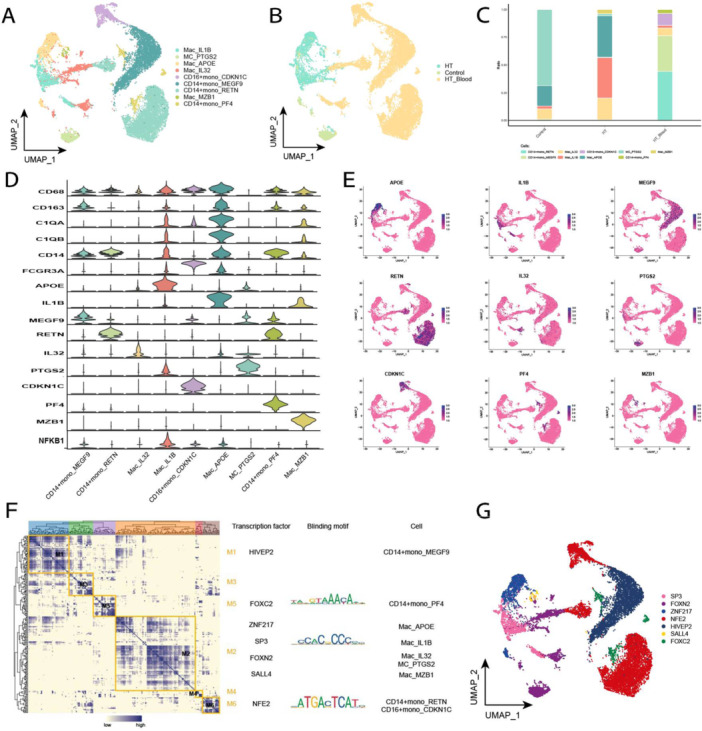
Single‐cell map of MC in HT. (A) Single‐cell profiles of MC subpopulations. Each color represents a different subpopulation. (B) Single‐cell profiles of MC based on sample origin. (C) Differences in abundance of MC subpopulations in different subgroups. (D) Expression levels of marker genes in each subpopulation. (E) Single‐cell profiles of marker gene expression in each subpopulation. (F) Co‐expression modules of transcription factors in MC of HT patients. Left: Identification of regulatory modules based on their CSI matrices; middle: binding patterns of representative transcription factors in modules; right: cell subpopulations where transcription factors are located. (G) UMAP single‐cell atlas mapping specific GRNs in MC subpopulations.

After re‐clustering the MCs according to the annotation method, nine cell subgroups were totally obtained (Figure 5A). MCs with a high expression level of CD14 in PBMCs of HT patients were classified as classical CD14^+^ monocytes (CD14^+^mono), while MCs that abundantly expressed FCGR3A (CD16) were labeled as nonclassical CD16^+^ monocytes (CD16^+^mono) [19]. Changes in abundance of these subgroups were displayed in a single‐cell atlas (Figure 5B). The most significant changes in the Mac_APOE and Mac_IL1B subpopulations in tissues of HT patients were noted (Figure 5C). This suggests that the expression profiles of APOE and IL1B genes may be implicated in the development of HT. Additionally, the marker genes of macrophage subsets were specifically expressed in the corresponding specific subsets (Figure 5D,E). In addition, GRNs of MC subpopulations were constructed, and the regulatory factors were hierarchically clustered according to CSI values. It was found that TFs were categorized into six modules in the GRNs of SP3, FOXC2, NFE2, and ZNF217 (Figure 5F), in turn regulating the expression levels of MCs‐specific genes in HT tissue (Figure 5G).

### 3.5 The Role of TFCs in HT

**Figure 6:**
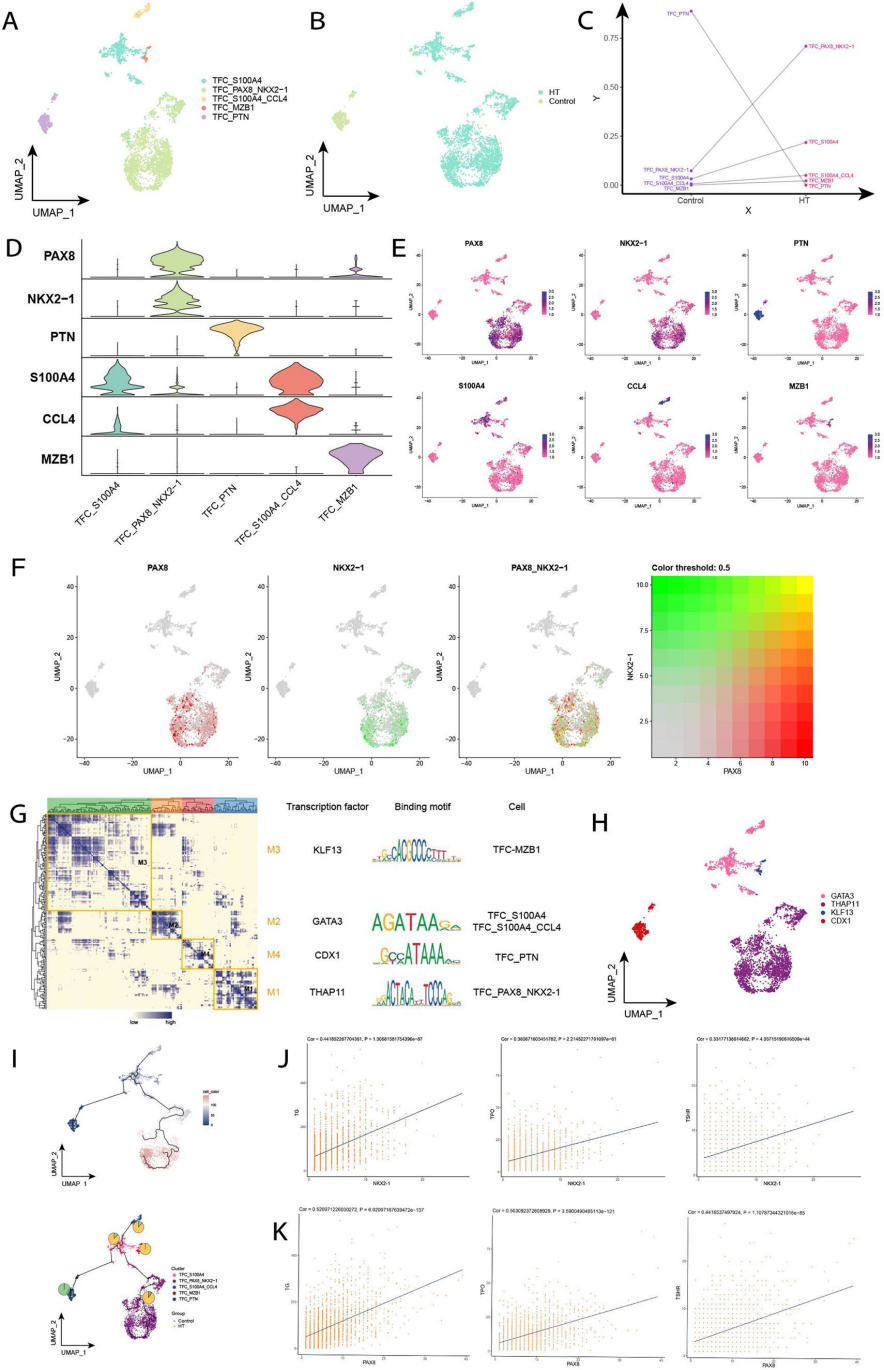
Single‐cell map of TFC in HT. (A) Single‐cell profiles of TFC subpopulations. Each color represents a different subpopulation. (B) Single‐cell profiles of TFC based on sample source. (C) Differences in abundance of TFC subpopulations in different subgroups. (D) Expression levels of marker genes in each subpopulation. (E) Single‐cell profiles of marker gene expression in each subpopulation. (F) Single‐cell profiles indicating co‐expression of PAX8 gene in relation to NKX2‐1 gene. (G) TFCs of HT patients in co‐expression module of transcription factors. Left: Identification of regulatory modules based on their CSI matrices; middle: binding patterns of representative transcription factors in modules; right: cell subpopulations where transcription factors are located. (H) UMAP single‐cell atlas plots GRNs specific to subpopulations of TFCs. (I) Temporal analysis indicating chronological values of TFCs from control to disease progression; single‐cell atlas‐pie chart plots illustrating temporal trajectory of TFCs from control to disease progression, in which the pie charts represent the proportion of HT and control in each subpopulation. (J) It displays the correlation of NKX2‐1 expression level with Tg, TPO, and TSHR calculated cyclically in the single‐cell atlas. (K) It illustrates the correlation of PAX8 expression level with Tg, TPO, and TSHR calculated cyclically in the single‐cell atlas.

Division of TFCs into five subpopulations (Figure 6A) enabled the construction of single‐cell profiles based on sample sources (Figure 6B). The results revealed that the abundance of TFC_PTN subpopulation was significantly elevated in a healthy control's thyroid tissues, and the abundance of TFC subpopulation with double‐positive PAX8 and NKX2‐1 genes was significantly risen in HT patients' tissues (Figure 6C). The violin and single cell mapping maps showed specific high expression of TFC subgroup marker genes (Figure 6D,E). Co‐expression of these two genes, PAX8 and NKX2‐1, was also found (Figure 6F), leading to the hypothesis that they could be involved in HT by influencing the function of TFCs in the pathogenesis of HT. With TFs as the hub, GRN was divided into four modules (KLF13, GATA3, CDX1, THAP11) to regulate the expression level of specific genes in TFC subsets of thyroid tissue of HT patients (Figure 6G,H). Pseudotime analysis of the developmental trajectory of TFCs was found to start from TFC_PTN and move toward TFC_S100A4 before eventually differentiating into three end‐states of TFC_S100A4_CCL4, TFC_MZB1, and TFC_PAX8_NKX2‐1 (Figure 6I).

Finally, we summarize the main results of this paper in the following table. (Supporting Information S1: Table 3).

## 4 Discussion

HT is a chronic inflammatory disease affecting thyroid tissue [20]. Its development is primarily attributed to a loss of immune tolerance, influenced by both genetic and environmental factors [21]. Antigen‐presenting cells play a crucial role by presenting antigens to T cells, which in turn activate B cells to produce a significant amount of TPOAb and TgAb autoantibodies. These autoantibodies contribute to the destruction of TFCs. Additionally, the presence of autoantibodies leads to the infiltration of thyroid tissue by lymphocytes, driven by a variety of inflammatory factors initiated by both humoral and cellular immune responses [22]. This cascade results in considerable inflammation and apoptosis of thyroid tissue, ultimately leading to tissue destruction and thyroid dysfunction [23]. Our research aims to explore the interactions between TFCs and immune cells throughout the inflammatory process at the cellular level (Supporting Information S1: Table 3).

In constructing single‐cell profiles for T cells, T cells expressing the marker genes (IL2RA or FOXP3) were annotated as regulatory T (Treg) cells. However, in this study, Treg cells were not found in the profiles as none of the cell subpopulations in T cells expressing IL2RA or FOXP3. This could be attributed to the limited number of Treg cells in the samples of this study, indicating that single‐cell 10× sequencing failed to detect them.

NKT can mediate cytotoxic activity and secrete cytokines in response to immune stimulation [24]. Killer cell lectin‐like receptor D1 (KLRD1), also known as CD94, is an antigen that is preferentially expressed in NK cells, and it is classified as a type‐II membrane protein due to its external C‐terminus. Killer cell lectin‐like receptor C2 (KLRC2), also known as NKG2, is mainly expressed in NK cells and encodes a family of transmembrane proteins characterized by type‐II membrane orientation (extracellular C‐terminus) and the presence of C‐type lectin structural domains. KLRD1_KLRC2 has been identified as an immune‐activating receptor [25], recognizing HLA‐E of the nonclassical major histocompatibility complex (MHC) class Ib on a subset of cytotoxic lymphocytes. Noel R Rose in his article mentions that the MHC was involved in the initial immune response to thyroglobulin [26]. In the present study, an increased abundance of a subpopulation of NKT cells expressing KLRD1_KLRC2 positivity was identified in the PBMCs of HT patients. It is hypothesized that KLRD1_KLRC2 may contribute to the development of HT by recognizing MHC to trigger the immune response, thereby recruiting more inflammatory cells in the PBMCs, which may infiltrate thyroid tissues and destroy TFCs.

The infiltration of B cells is crucial for the development of HT. This study has identified a novel subgroup of B cells, referred to as B_MEF2B_BCL6. Myocyte enhancer binding factor 2B (MEF2B) is a member of the myocyte enhancer factor 2 (MEF2) transcription factor family. Previous research in diffuse large B‐cell lymphoma has shown that MEF2B directly activates the transcription of BCL6 in germinal center (GC) B cells [27]. BCL6 plays a vital role in the formation and maintenance of GCs. Studies indicate that the absence of BCL6 can hinder the formation of GCs and the production of high‐affinity antibodies, underscoring its central importance in regulating high‐affinity antibody production [28]. Furthermore, investigations into autoimmune thyroid diseases have revealed that GCs predominantly form within the thyroid tissue of HT patients [29]. In the context of HT, B cells are activated with the assistance of T cells, leading to their proliferation and the formation of germinal centers composed primarily of actively dividing B cells. Ultimately, fully activated B cells in the GC differentiate into plasma and memory B cells that secrete specific antibodies. Our findings indicate that the B_MEF2B_BCL6 subgroup is enriched in the HT patient group. MEF2B appears to facilitate the formation of GCs in HT by activating BCL6 transcription, thus promoting the rapid proliferation and differentiation of B cells into plasma cells. This mechanism significantly enhances the inflammatory response associated with HT.

The present study indicated that Ig‐producing plasma cells were notably enriched in B‐cell subpopulations, a prominent characteristic of HT as an organ‐specific autoimmune disease, marked by the elevated production of high titers of TgAb and TPOAb. These TgAb and TPOAb were identified as being of immunoglobulin (Ig) origin, aligning with the findings of the present study. Moreover, Song Huaidong observed a significant presence of tissue‐generating center B cells and plasma cells, underscoring the thyroid as the primary source of autoimmune antibodies [8].

In the present study, it was revealed that the abundance of a subpopulation of macrophages expressing apolipoprotein E (APOE) was elevated in in thyroid tissues of HT patients. APOE is mainly synthesized by the liver, while it is also produced in other tissues, such as brain and macrophages. It primarily plays a regulatory role in lipoprotein metabolism, as it is involved in hepatic lipoprotein secretion, lipoprotein recycling, as well as being a high‐affinity ligand for cellular lipoprotein uptake [30]. APOE, a major component of very low‐density lipoproteins (VLDLs), has been shown to affect immune activation of antigen‐presenting cells by regulating plasma lipoprotein clearance [31]. Bouchareychas demonstrated this finding in a mouse model of APOE, in which it was indicated that macrophage exosomes could inhibit inflammatory responses through microRNA targeting of NF‐κB and tumor necrosis factor‐α (TNF‐α) signaling [32]. The activated NF‐κB contributes to the polarization of M1 macrophages, triggering a complex signaling cascade response, leading to the production of pro‐inflammatory cytokines, such as TNF‐α and interleukin‐1β (IL‐1β) [33]. The present study revealed that APOE‐positive macrophages were significantly expressed in HT patients' tissues and NF‐κB was also expressed in this subpopulation (Figure 5D). Consequently, it was hypothesized that APOE‐positive macrophage exosomes could target NF‐κB signaling via microRNA and activate NF‐κB signaling, polarizing M1 macrophages to trigger a cascade response. This, in turn, leads to massive inflammatory factors infiltrating thyroid cells, resulting in their destruction and contributing to the pathogenesis of HT.

IL‐1β is a potent pro‐inflammatory cytokine, playing a crucial role in the defense response to host infection and injury. It is produced and secreted primarily by monocytes and macrophages [34]. Macrophages/monocytes are also involved in the processing and presentation of thyroid autoantigens and in the mediation of cytotoxic responses [35]. Of these, interleukin‐1 (IL‐1) stimulates the proliferation of thyroid cells while inhibiting the synthesis and release of thyroid hormones. In addition, IL‐1 stimulates the production of other cytokines by the thyroid gland, thereby disrupting the thyroid epithelial barrier and affecting thyroid function [36]. Sun et al. [37] found that IL‐1β mRNA level was elevated in the thyroid tissue of HT patients, along with an increase in the localized monocyte infiltration of the thyroid tissue, which led to an increase in apoptosis of the gland. The major pathway leading to hypothyroidism is the Fas‐mediated apoptotic pathway. Fas in TFCs specifically binds to FasL on the surface of infiltrating lymphocytes within the thyroid gland, promoting apoptosis and leading to hypothyroidism. It has been reported that the high level of IL‐1β produced in the HT thyroid gland could induce the expression level of Fas in normal thyroid cells. Cross‐linking of Fas can lead to notable apoptosis of thyroid cells [38], which in turn leads to hypothyroidism, the main clinical symptom of HT. The present study indicated that the increased abundance of Mac_IL1B subpopulation in tissues of HT patients could be attributable to the high expression level of IL1B, which specifically binds to FasL, causing apoptosis of thyroid cells.

PAX8 is a transcription factor from the paired box domain family, expressed in various tissues including the fallopian tubes, seminal vesicles, epididymis, islet cells, and lymphocytes [39]. NKX2‐1, known as thyroid transcription factor‐1 (TTF‐1), is part of the NKX2 family and functions as a homeobox domain transcription factor found in the thyroid gland, lung tissue, basal ganglia neurons, cortical interneurons, and the hypothalamus [40]. Previous studies have demonstrated that NKX2‐1 enhances the expression of HLA Class I molecules and thyroid‐specific autoantigens (such as Tg and TPO) [41]. Both NKX2‐1 and HLA Class I antigens were found to be significantly elevated in patients with HT, and there is a positive correlation between NKX2‐1 and HLA Class I molecules [42]. HLA Class I molecules are critical for presenting autogenic peptides to immune cells and are associated with the pathogenesis of various organ‐specific autoimmune diseases, often representing one of the earliest signs of autoimmune attacks in these conditions [43, 44]. Our current study reveals a significant increase in the abundance of the TFC_PAX8_NKX2‐1 subgroup in the tissues of HT patients. Moreover, we observed co‐expression of NKX2‐1 and PAX8 in HT tissues, with correlation analysis indicating a positive relationship between NKX2‐1, PAX8, and thyroid autoantigens Tg, TPO, and TSHR. We hypothesize that TFCs may be induced to express HLA‐like molecules to present autoantigens to immune cells under the pathological conditions associated with HT. Additionally, NKX2‐1 and PAX8 may contribute to the upregulation of thyroid antigen expression, suggesting that various thyroid antigen peptides could be presented to immune cells, thereby activating cytotoxic immune responses.

HT is the most common autoimmune disorder, yet its symptoms frequently present as nonspecific. The diagnosis of HT primarily depends on a positive TPOAb test [45]. Recently, there has been a notable increase in the incidence of HT, which has placed significant demands on medical resources. Consequently, it is imperative to identify specific targets that can enhance the production of thyroid autoantibodies, facilitating more effective treatment strategies for HT. Using single‐cell sequencing, we discovered a novel subpopulation related to B cells and TFCs in HT that can stimulate the production of TPOAb through specific gene activation. Our future research will focus on inhibiting TPOAb production by targeting these identified genes, with the goal of advancing cause‐and‐effect‐based treatment strategies for HT. Additionally, we will explore combination therapies that address both inflammatory and immune responses to improve treatment outcomes. Finally, identifying reliable biomarkers to assess disease activity and predict prognosis will help in achieving earlier diagnoses and more targeted therapeutic strategies. This research may include investigating the role of existing anti‐inflammatory drugs in immunomodulatory therapies.

Although our research is based on scientific bioinformatics analytical methods, our study has several limitations. The sample of this study was relatively small. Though thyroid lesion tissues from two HT patients and one healthy control were obtained from our hospital for single‐cell transcriptomic sequencing, only four thyroid tissues and PBMCs were obtained from the database. Further expanding the sample range and molecular and cellular experiments in our subsequent article will validate our findings and provide deeper insights into the molecular interactions.

In conclusion, this study used single‐cell transcriptomic sequencing to investigate the pathogenesis of HT at the cellular level, to provide further efficacious therapeutic targets for HT.

## Author Contributions

J.Z.: conceptualization, methodology. W.Y.: data collection, data analysis, writing‐review and graphing. W.Y. and W.X.: data analysis, writing. W.Y., S.K., and W.Y.: data analysis, graphing. J.Z., M.Y., and T.Y.: Conceptualization, supervision, writing‐review and editing.

## Consent

All patients provided written informed consent.

## Conflicts of Interest

The authors declare no conflicts of interest.

## Supporting information

Supporting information.

## Data Availability

Single‐cell sequencing data generated in this study have been deposited in the Genome Sequence Archive in the National Genomics Data Center, China National Center for Bioinformation/Beijing Institute of Genomics, Chinese Academy of Sciences, GSA‐Human access number: GSA‐Human access number: HRA001684 and HRA002138.
