# Supplementary material for: Construction of the Single‐Cell Landscape of Hashimoto's Thyroiditis Tissue and Peripheral Blood by Single‐Cell RNA Sequencing
Source: Immun Inflamm Dis. 2025 Feb 11;13(2):e70153. doi: 10.1002/iid3.70153 (PMC11811715; doi:10.1002/iid3.70153)
Supplement: Supplementary file 1 — Supporting information. [file IID3-13-e70153-s001.docx]

**Supplementary Table 1. Sampling and sequencing the clinical data of the research subjects.**

| Sample | Sex | Age | Diagnosis | TSH (0.27-4.2  mIU/L) | FT4  (12-22  pmol/L) | FT3  (2.8-7.1  pmol/L) | Tg-Ab (0-115  IU/ml) | TPO-Ab  (0-34  IU/ml) |
| --- | --- | --- | --- | --- | --- | --- | --- | --- |
| N1 | F | 49 | Thyroid adenoma | 2.62 | 14.44 | 5.13 | 16.39 | 7.28 |
| HT1 | F | 58 | PTC&HT | 1.59 | 14.7 | 3.48 | 1352 | 262.8 |
| HT2 | F | 56 | PTC&HT | 2.54 | 15.43 | 4.41 | 362.8 | 422.4 |

F female, PTC papillary thyroid carcinoma, HT Hasimoto's thyroiditis, TSH thyroid stimulating hormone, FT4 free thyroxine, FT3 free thiiodothyronine, Tg-Ab thyroglobulin antibodies, TPO-Ab thyroperoxidase antibodies

**Supplementary Table 2. Clinical data of study subjects in the GEO database.**

| Sample | Sex | Age | Diagnosis | TSH (0.34-5.6  uIU/ml) | FT4  (0.58-1.46  ng/dl) | FT3  (2.5-3.9  pg/ml) | Tg-Ab (0-115  IU/ml) | TPO-Ab  (0-9  IU/ml) |
| --- | --- | --- | --- | --- | --- | --- | --- | --- |
| HT12 | F | 43 | Thyroid nodule  &HT | 1.62 | 0.73 | 2.95 | 401 | 97.4 |
| HT13 | F | 61 | PTC&HT | 1.95 | 0.77 | 3.62 | 610.4 | 46.4 |
| HT14 | F | 60 | PTC&HT | 4.94 | 0.92 | 3.89 | 875.2 | 6.7 |
| HT28 | F | 60 | Thyroid nodule  &HT | 6.05 | 0.9 | 3.27 | >4000 | 7.5 |
| HT29 | F | 31 | PTC&HT | 1.28 | 0.96 | 3.2 | 1229 | 0.6 |

F female, PTC papillary thyroid carcinoma, HT Hasimoto's thyroiditis, TSH thyroid stimulating hormone, FT4 free thyroxine, FT3 free thiiodothyronine, Tg-Ab thyroglobulin antibodies, TPO-Ab thyroperoxidase antibodies

**Supplementary Table 3.** **Mechanism of action of different cell types in HT.**

| Cell Types Tissue Gene | The findings in this article |
| --- | --- |
| NKT PBMC KLRD1 and KLRC2 | recognize MHC to trigger the immune response; recruit more inflammatory cells to infiltrate thyroid tissues and destroy TFCs. |
| B cells Thyroid MEF2B and BCL6 | contribute to the formation of GCs in thyroid tissue. |
| Mac Thyroid APOE | activate NF-κB signaling; polarize M1 macrophages to trigger a cascade response. |
| Mac Thyroid IL1B | apoptosis of TFCs through activation of the Fas-FasL pathway |
| TFC Thyroid PAX8 and NKX2-1 | NKX2-1 and PAX8 were positively correlated with Tg, TPO, and TSHR |
